# Liver Transplantation for Metabolic Dysfunction-Associated Steatotic Liver Disease after Pancreaticoduodenectomy

**DOI:** 10.70352/scrj.cr.25-0264

**Published:** 2025-09-06

**Authors:** Takeshi Kano, Ryugen Takahashi, Nobuhisa Akamatsu, Yujiro Nishioka, Yuichiro Mihara, Akihiko Ichida, Takeshi Takamoto, Yoshikuni Kawaguchi, Kiyoshi Hasegawa

**Affiliations:** Artificial Organ and Transplantation Surgery Division, Department of Surgery, Graduate School of Medicine, The University of Tokyo, Tokyo, Japan

**Keywords:** liver transplantation, metabolic dysfunction-associated steatotic liver disease/steatohepatitis, pancreaticoduodenectomy

## Abstract

**INTRODUCTION:**

Steatotic liver disease (SLD) may develop in some patients after pancreaticoduodenectomy (PD), but no cases requiring liver transplantation (LT) have been reported to date. Here, we present two cases in which LT was performed for decompensated liver cirrhosis (LC) after PD.

**CASE PRESENTATION:**

Case 1 was a 53-year-old man with obesity, metabolic-associated SLD (MASLD), and diabetes mellitus. The patient underwent PD for an intraductal papillary mucinous neoplasm. His liver function worsened and he developed decompensated LC 6 years later, eventually requiring LT. Due to poor mobility of the jejunal limb caused by severe adhesions and the presence of a pancreatojejunostomy, a choledochojejunostomy was performed at the more distal site of the common bile duct than usual. He developed hemobilia and biliary leakage but was discharged on POD 107. Liver function has been good for 2 years after LT without MASLD recurrence, although endoscopic treatment is periodically required for biliary stricture. Case 2 was a 46-year-old man with obesity, SLD, and a history of excessive alcohol consumption. The patient underwent PD for duodenal cancer. Five years later, he developed decompensated LC, which required living-donor LT. For biliary reconstruction, a new jejunal limb was created and elevated. He was discharged on POD 79. He has repeatedly developed cholangitis, but his liver function has been good for 6 years without SLD recurrence.

**CONCLUSIONS:**

Steatohepatitis can worsen following PD and may lead to decompensated LC, ultimately requiring LT. Therefore, screening for steatohepatitis and its risk factors prior to PD is essential, and prophylaxis should be considered. LT after PD presents surgical challenges and biliary reconstruction with some procedural modifications.

## Abbreviations

BMI
: body mass index
CBD
: common bile duct
DM
: diabetes mellitus
IPMN
: intraductal papillary mucinous neoplasm
LC
: liver cirrhosis
LHA
: left hepatic artery
LT
: liver transplantation
MASLD
: metabolic associated steatotic liver disease
MELD
: model for end-stage liver disease
PD
: pancreaticoduodenectomy
RTBD
: retrograde transhepatic biliary drainage
SLD
: steatotic liver disease
TNM
: tumor-node-metastasis
UICC
: Union for International Cancer Control

## INTRODUCTION

MASLD is a common complication in the late postoperative period after PD and total pancreatectomy, with a reported incidence of up to 37.0% after PD.^1)^ Cases of decompensated LC requiring LT have not been reported. Here, we present two cases in which LT was performed for decompensated LC due to worsening SLD following PD.

## CASE PRESENTATION

### Case 1

**Fig. 1:**
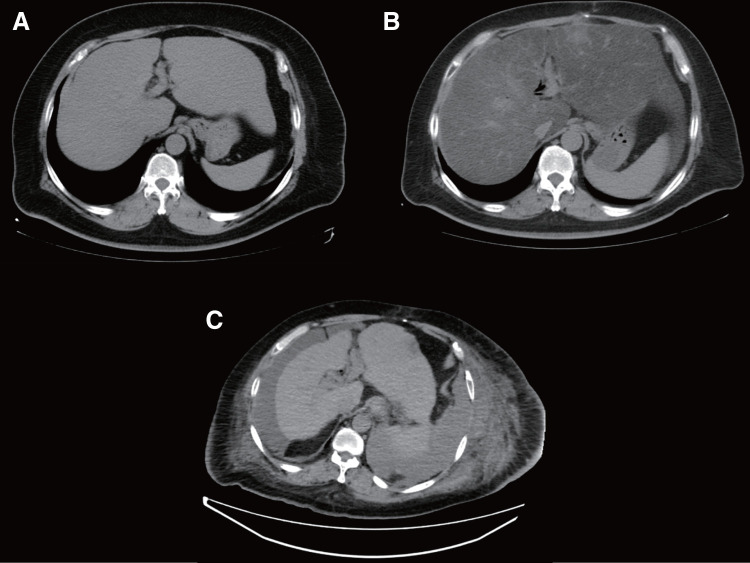
Chronologic changes in CT findings in Case 1. (**A**) CT before PD showed hepatic steatosis. The CT value of the liver was 40 HU. (**B**) CT 6 months after PD showed progression of hepatic steatosis. The CT value was −2 HU. (**C**) CT before liver transplantation showed an atrophic liver with an irregular surface and blunt edge, consistent with complete liver cirrhosis. The CT value increased to 40 HU, indicating progression of liver fibrosis. PD, pancreaticoduodenectomy; HU, Hounsfield units

A 53-year-old man with obesity (BMI 38.5), MASLD, well-controlled DM without medications, and hypertension—but without hyperlipidemia—was diagnosed with a branch duct IPMN based on CT scanning to evaluate a pancreatic head mass detected by ultrasound sonography at a regular checkup. An enhancing solid component was observed within the IPMN and subtotal stomach-preserving PD with modified Child’s reconstruction was performed at another hospital 5 months after the diagnosis. The pathologic diagnosis was branch duct IPMN with noninvasive cancer. According to the 7th edition of the UICC TNM system,^2)^ the pathological stage was TisN0M0 (Stage 0). No recurrence of the cancer was detected during follow-up, but a contrast-enhanced CT scan taken 6 months after PD demonstrated a sharp decrease in CT value of the liver, indicating progression of hepatic steatosis (**Fig. 1A**, **1B**). The liver parenchyma became atrophic over time (**Fig. 1C**) and his liver function gradually worsened. Pancrelipase was not administered during that period. No worsening of DM was observed after PD. The patient’s BMI remained around 33.

**Fig. 2:**
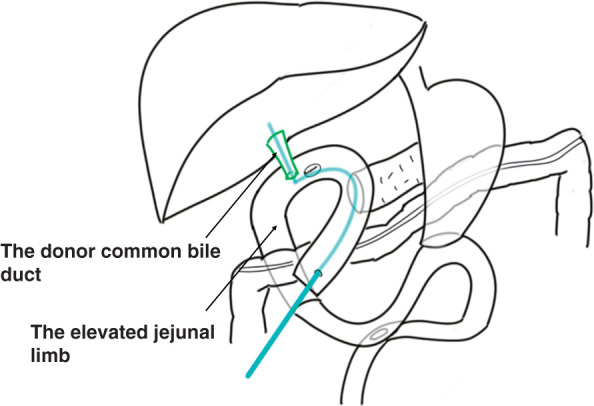
Biliary reconstruction in Case 1. The donor CBD is shown in green color. The extremely poor mobility of the jejunal limb caused by severe adhesions and distal fixation at the pancreaticojejunostomy required enough length of the donor CBD. Choledochojejunostomy was successfully created by the anastomosis between the donor CBD at the more distal site than usual and a newly created hole of the jejunal limb nearby the original hole. A retrograde RTBD tube was placed as an externalized stent, which is shown in light blue color. CBD, common bile duct; RTBD, retrograde transhepatic biliary drainage

**Fig. 3:**
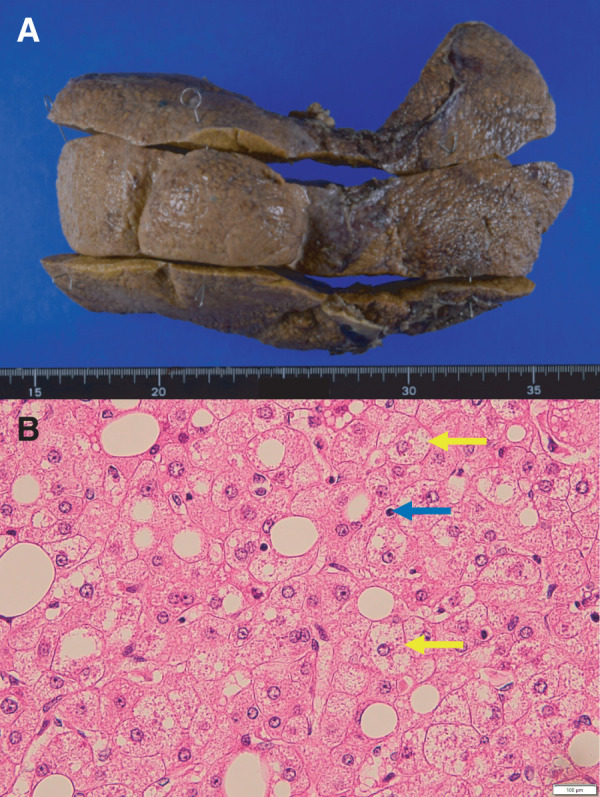
Macroscopic appearance and pathologic examination of the resected specimen. (**A**) The resected liver showed atrophy, surface nodularity, and contour irregularity. (**B**) Ballooning hepatocytes (yellow arrows) and inflammatory cell infiltration (blue arrow) were consistent with metabolic dysfunction-associated steatohepatitis (hematoxylin and eosin staining, ×400).

Six years later, the patient developed decompensated LC with jaundice, refractory ascites, and pleural effusion. At the same time, he was also diagnosed with hepatorenal syndrome based on the trend of his renal function, which had been normal but deteriorated in parallel with his liver function. At the time of listing for deceased-donor LT, laboratory tests showed jaundice (total bilirubin 6.5 mg/dL), coagulopathy (prothrombin time-international normalized ratio 1.4), hypoalbuminemia (albumin 2.1g/dL), and renal failure (creatinine 5.38 mg/dL). The MELD score was 32. Deceased-donor LT was performed one month after his listing, when he was 60 years of age. The donor was a 57-year-old man who died from subarachnoid hemorrhage. Due to severe intraabdominal adhesions, a long time was required for dissection of the jejunal limb, the LHA, and main portal vein, and massive bleeding occurred. The right hepatic artery could not be identified or encircled, and the LHA was anastomosed to the donor common hepatic artery. The extremely poor mobility of the jejunal limb caused by severe adhesions and distal fixation at the pancreaticojejunostomy required enough length of the donor CBD for biliary reconstruction. Consequently, a choledochojejunostomy was successfully created by the anastomosis between the donor CBD at the more distal site than usual and a newly created hole of the jejunal limb nearby the original hole which was closed with sutures. A 2.5-mm RTBD tube was placed as an externalized stent (**Fig. 2**), which was removed on POD 71. The operation time and cold/warm ischemic time were 560 min and 358/40 min, respectively. Estimated blood loss was 43950 mL. Macroscopically, the resected liver showed atrophy, surface nodularity, and contour irregularity. Pathologic examination showed ballooning degeneration, inflammatory cell infiltration, and lipid deposition in approximately 10% of hepatocytes, consistent with LC caused by metabolic dysfunction-associated steatohepatitis (MASH) (**Fig. 3**). Postoperatively, the patient developed hemobilia and a bile leak requiring both endoscopic and percutaneous biliary drainage, maybe related with the longer preservation of CBD, but was successfully managed and discharged on POD 107. Pancrelipase has been administered postoperatively. Hyperglycemia was observed after the initiation of postoperative steroid therapy, and oral hypoglycemic agents were temporarily administered. The patient has been followed up regularly on an outpatient basis, and glycemic control has remained stable without any medication. The patient has been receiving continuous dietary counseling from a registered dietitian. His latest BMI showed a decrease to 29.9. His liver function has been good for 2 years after deceased-donor LT without MASLD recurrence, although periodical endoscopic treatment has been applied for anastomotic biliary strictures.

### Case 2

A 46-year-old man with obesity (BMI 31.1) and hypertension—but without DM and hyperlipidemia—was diagnosed with duodenal cancer during a workup for anemia. He had a history of excessive alcohol consumption (100 g per day) and developed steatohepatitis. Laboratory tests showed elevated liver enzymes (aspartate aminotransferase 59 U/L, alanine aminotransferase 75U/L). Total bilirubin (0.4 mg/dL), prothrombin time-international normalized ratio (0.86), and albumin levels (3.6 g/dL) were within normal limits. Subtotal stomach-preserving PD with Roux-en-Y reconstruction was performed at our hospital, and the pathologic diagnosis was duodenal cancer. According to the 7th edition of the UICC TNM system,^2)^ the pathological stage was T3N0M0 (Stage IIA). At the time of PD, the steatohepatitis had not yet progressed to LC and pancrelipase was started to prevent aggravation. No recurrence was detected for 5 years after the surgery. Glycemic control remained stable in the postoperative period. The patient’s BMI remained over 30. The patient’s alcohol consumption (28 g per day) continued and his liver function gradually worsened. Eventually, the patient developed decompensated LC and the MELD score was 32. Laboratory tests showed jaundice (total bilirubin 5.1 mg/dL), coagulopathy (prothrombin time-international normalized ratio 1.8), and hypoalbuminemia (albumin 2.7g/dL). His renal function was normal (creatinine 0.73 mg/dL). He was strongly advised to abstain from alcohol.

**Fig. 4:**
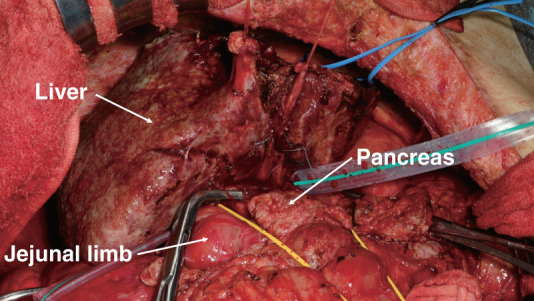
Intraabdominal findings at the time of liver transplantation in Case 2. The hepatic hilum was difficult to divide due to severe adhesions so total hepatectomy with *en bloc* hilar dissection was performed.

**Fig. 5:**
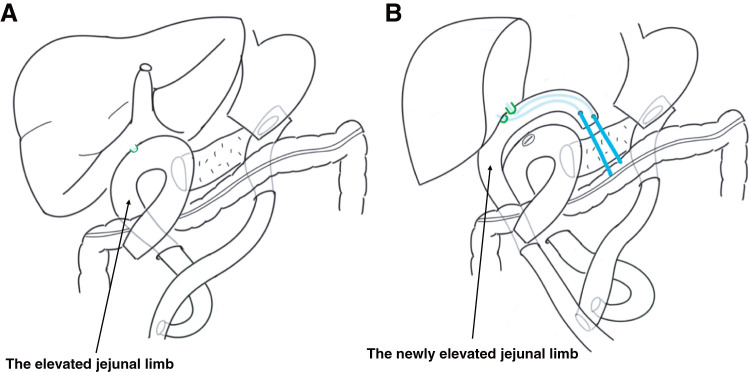
Biliary reconstruction in Case 2. (**A**) Roux-en-Y reconstruction at the time of pancreaticoduodenectomy. The CBD is shown in green color. (**B**) The existing jejunal limb could not be mobilized, so the donor bile ducts, which are shown in green color were anastomosed to the newly elevated jejunal limb. An RTBD tube was placed as an externalized stent, which is shown in light blue color. CBD, common bile duct; RTBD, transhepatic biliary drainage

After 14 months of abstinence, which met a mandatory requirement of “no less than 6 months of abstinence” for living-donor LT at our hospital, ABO-incompatible living-donor LT with a right liver graft was performed when he was 51 years of age. The donor was the patient’s wife. She was 51 years of age with a BMI of 21.5. Adhesions around the hepatic hilum were too severe to perform a safe hilum dissection. Therefore, *en bloc* hilar dissection was performed under Pringle’s maneuver, and the portal vein and hepatic arteries were dissected after total hepatectomy (**Fig. 4**). In this case, the original jejunal limb was deemed inappropriate for biliary reconstruction, and the right hepatic duct was anastomosed to a newly elevated jejunal limb using a 2.0-mm RTBD tube as an externalized stent (**Fig. 5**), which was removed on POD 173. The operation time and cold/warm ischemic time were 901 min and 112/45 min, respectively. Estimated blood loss was 18065 mL. Macroscopically, the resected liver exhibited atrophy, surface nodularity, and contour irregularity. Pathologic examination showed inflammatory cell infiltration, lipid deposition in less than 10% of hepatocytes, and relatively small pseudolobules compatible with LC caused by SLD. Postoperatively, the patient developed acute rejection and intra-abdominal infection but was discharged on POD 79. The patient has abstained from alcohol and his liver function has been good for 7 years after the living-donor LT without SLD recurrence. Hyperglycemia was observed after the initiation of postoperative steroid therapy; however, it was successfully controlled with dietary management alone. The patient has been followed up regularly on an outpatient basis, and glycemic control has remained stable without any medication. His most recent BMI decreased to 24.5.

## DISCUSSION

We present two cases requiring LT for decompensated LC secondary to SLD that progressed following PD. MASLD is a relatively common complication after PD and there is one case report of a patient who developed decompensated LC.^3)^ No cases requiring LT for decompensated LC after PD, however, have been reported to date. To the best of our knowledge, this is the 1st report describing such cases.

Unlike typical MASLD associated with metabolic syndrome, MASLD after PD is characterized by nonobesity, nonmetabolic syndrome, and a severe nutritional disorder,^4–7)^ and is associated with postoperative weight loss.^8,9)^ The cause of MASLD after PD is thought to be malnutrition due to exocrine pancreatic insufficiency, which reduces plasma levels of apoprotein B, suggesting impaired hepatic export of triglyceride.^5,10)^ Insufficient secretion of insulin after PD enhances peripheral lipolysis and increases hepatic-free fatty acid uptake.^5)^ Considering these underlying mechanisms, preoperative obesity and DM are considered as risk factors for MASLD, and preexisting SLD could worsen following PD. In fact, preoperative obesity is a reported risk factor for MASLD after PD.^11)^ Patients in Cases 1 and 2 were markedly obese and had been diagnosed with SLD prior to PD. Reduction of the pancreatic parenchyma by PD might have accelerated the progression of SLD to decompensated LC.

This report highlights the need for preoperative screening for SLD and its risk factors before PD. For high-risk patients, preoperative treatments such as reducing body weight or controlling DM are important. After surgery, factors aggravating MASLD should be managed and prophylaxis, such as pancrelipase, should be routinely administered.^3,6,12)^ In Case 1, the indication for PD was IPMN and the time from diagnosis to surgery was 5 months at the previous hospital. Further weight loss during that waiting period could have prevented MASLD aggravation. In addition, postoperative pancrelipase should have been administered and more aggressive weight reduction might have been beneficial in preventing disease progression. On the contrary, in Case 2, the waiting period before surgery was short and postoperative pancrelipase was started after the operation. However, the patient continued to consume 28 g of alcohol per day after PD. This amount of alcohol intake alone is unlikely to cause liver cirrhosis. But considering that even modest alcohol consumption is associated with less improvement in MASLD,^13)^ the patient should have completely abstained from alcohol. The combination of exocrine pancreatic insufficiency caused by PD, obesity, and continued alcohol intake ultimately led to the development of LC.

Notably, Cases 1 and 2 had SLD at the time of PD, and both underwent PD for noninvasive pancreatic cancer or non-pancreatic cancer at around the age of 50 years. In addition, they developed decompensated LC around 5 years after PD. These facts suggest that young patients with SLD who undergo PD for low malignant tumors are more likely to develop decompensated LC because they are expected to have a relatively longer remaining lifespan. Indeed, younger age is a recognized risk factor for MASLD after PD.^14)^ Although pancreatic cancer requiring massive resection of the pancreatic parenchyma is another risk factor for MASLD,^1,8,9)^ patients with non-pancreatic cancer may have a higher risk for developing LC than patients with pancreatic cancer.

The recurrence rate of MASLD after LT for MASH ranges from 4% to 33%,^15)^ and the rate is expected to be higher in LT recipients after PD. Generally, the cornerstone of preventing MASLD recurrence after LT is the effective management of risk factors such as DM, hypertension, dyslipidemia, and obesity.^16–18)^ Both Cases 1 and 2 presented with high obesity, making weight reduction essential, but at the same time, it was necessary to ensure adequate nutritional support considering the mechanisms underlying the development of MASH following PD, as described above.^4–7)^ In Case 1, pancrelipase was initiated after transplantation, and nutritional counseling was provided, which contributed to weight loss. In Case 2, the patient received guidance on alcohol abstinence and weight reduction. In both cases, hyperglycemia induced by postoperative steroid therapy was strictly managed with appropriate glycemic control. Fortunately, neither case has yet developed a recurrence of SLD after LT, but close monitoring of their liver function is essential.

LT surgery following PD is technically challenging. First, adhesions of the abdominal cavity, especially around the hilum of the liver, are severe after PD and require meticulous adhesiolysis, which can cause massive bleeding from the abdominal serosa. Second, careful dissection and preservation of the portal vein and hepatic arteries are critical. Finally, in addition to the absence of an extrahepatic bile duct after PD, biliary reconstruction is difficult due to the poor mobility of the jejunal limb. Similar to our experience, John A. Stauffer et al. reported that dense adhesions made dissection and reconstruction technically challenging based on their own cases of LT following pancreatic resection for metastatic liver tumors.^19)^ In particular, in a case of LT after PD, they failed to dissect the jejunal limb and stated that salvage of the intact pancreaticojejunostomy was potentially impossible. An intraoperative mortality was also reported during LT following pancreatic resection.^20)^

In our two cases, the original pancreaticojejunostomy was successfully preserved and the biliary system was reconstructed with careful dissection and surgical modification. In Case 1, a longer-than-usual donor CBD had to be preserved. Insufficient arterial blood supply to the lower CBD of the graft might have contributed to the development of biliary complications. In Case 2, because the graft was a right liver graft from a living donor, the length of the right hepatic duct was short, and another jejunal limb was newly elevated for the hepaticojejunostomy. Both cases developed biliary complications after LT and Case 1 requires periodic endoscopic treatment, but liver function has been good so far. Making some adjustments in the biliary reconstruction is an essential part of LT after PD.

## CONCLUSIONS

SLD can worsen to LC after PD, which ultimately requires LT. It is important to screen for steatohepatitis or its risk factors prior to PD and to take preventive measures. In addition, special attention must be paid to young patients with SLD who undergo PD for benign or low-grade malignancy. The LT procedure itself is challenging, and biliary reconstruction requires some modifications during LT after PD.

## DECLARATIONS

### Availability of data and materials

The datasets supporting the conclusions of this article are included within the article and its additional files.

## References

[1] Kato H, Isaji S, Azumi Y, et al. Development of nonalcoholic fatty liver disease (NAFLD) and nonalcoholic steatohepatitis (NASH) after pancreaticoduodenectomy: proposal of a postoperative NAFLD scoring system. J Hepatobiliary Pancreat Sci 2010; 17: 296–304. 19809782 10.1007/s00534-009-0187-2

[2] Sobin LHGM, Wittekind C, ed. TNM classification of malignant tumours. 7 ed. 2009, Wiley-Blackwell: Oxford.

[3] Sim EH, Kwon JH, Kim SY, et al. Severe steatohepatitis with hepatic decompensation resulting from malnutrition after pancreaticoduodenectomy. Clin Mol Hepatol 2012; 18: 404–10. 23323257 10.3350/cmh.2012.18.4.404 PMC3540378

[4] Jeon D, Park BH, Lee HC, et al. The impact of pylorus preservation on the development of nonalcoholic fatty liver disease after pancreaticoduodenectomy a historical cohort study. J Hepatobiliary Pancreat Sci 2022; 29: 863–73. 35434927 10.1002/jhbp.1150

[5] Kang CM, Lee JH. Pathophysiology after pancreaticoduodenectomy. World J Gastroenterol 2015; 21: 5794–804. 26019443 10.3748/wjg.v21.i19.5794 PMC4438013

[6] Murata Y, Mizuno S, Kato H, et al. Nonalcoholic steatohepatitis (NASH) after pancreaticoduodenectomy: association of pancreatic exocrine deficiency and infection. Clin J Gastroenterol 2011; 4: 242–8. 26189528 10.1007/s12328-011-0226-9

[7] Satoh D, Yagi T, Nagasaka T, et al. CD14 upregulation as a distinct feature of non-alcoholic fatty liver disease after pancreatoduodenectomy. World J Hepatol 2013; 5: 189–95. 23671723 10.4254/wjh.v5.i4.189 PMC3648650

[8] Izumi H, Yoshii H, Fujino R, et al. Factors contributing to nonalcoholic fatty liver disease (NAFLD) and fat deposition after pancreaticoduodenectomy: a retrospective analysis. Ann Gastroenterol Surg 2023; 7: 793–9. 37663962 10.1002/ags3.12673 PMC10472401

[9] Tanaka N, Horiuchi A, Yokoyama T, et al. Clinical characteristics of de novo nonalcoholic fatty liver disease following pancreaticoduodenectomy. J Gastroenterol 2011; 46: 758–68. 21267748 10.1007/s00535-011-0370-5

[10] Tanaka N, Takahashi S, Fang ZZ, et al. Role of white adipose lipolysis in the development of NASH induced by methionine- and choline-deficient diet. Biochim Biophys Acta Mol Cell Biol Lipids 2014; 1841: 1596–607. 10.1016/j.bbalip.2014.08.015 PMC4188754 25178843

[11] Kato H, Kamei K, Suto H, et al. Incidence and risk factors of nonalcoholic fatty liver disease after total pancreatectomy: a first multicenter prospective study in Japan. J Hepatobiliary Pancreat Sci 2022; 29: 428–38. 34863034 10.1002/jhbp.1093

[12] Nagai M, Sho M, Satoi S, et al. Effects of pancrelipase on nonalcoholic fatty liver disease after pancreaticoduodenectomy. J Hepatobiliary Pancreat Sci 2014; 21: 186–92. 23798362 10.1002/jhbp.14

[13] Ajmera V, Belt P, Wilson LA, et al. Among patients with nonalcoholic fatty liver disease, modest alcohol use is associated with less improvement in histologic steatosis and steatohepatitis. Clin Gastroenterol Hepatol 2018; 16: 1511–1520 e5. 29378307 10.1016/j.cgh.2018.01.026 PMC6098737

[14] Sato R, Kishiwada M, Kuriyama N, et al. Paradoxical impact of the remnant pancreatic volume and infectious complications on the development of nonalcoholic fatty liver disease after pancreaticoduodenectomy. J Hepatobiliary Pancreat Sci 2014; 21: 562–72. 24824077 10.1002/jhbp.115

[15] Patil DT, Yerian LM. Evolution of nonalcoholic fatty liver disease recurrence after liver transplantation. Liver Transpl 2012; 18: 1147–53. 22740341 10.1002/lt.23499

[16] Burke A, Lucey MR. Non-alcoholic fatty liver disease, non-alcoholic steatohepatitis and orthotopic liver transplantation. Am J Transplant 2004; 4: 686–93. 15084161 10.1111/j.1600-6143.2004.00432.x

[17] Vallin M, Guillaud O, Boillot O, et al. Recurrent or de novo nonalcoholic fatty liver disease after liver transplantation: natural history based on liver biopsy analysis. Liver Transpl 2014; 20: 1064–71. 24961607 10.1002/lt.23936

[18] Mehtani R, Rathi S. Recurrence of primary disease after adult liver transplant - risk factors, early diagnosis, management, and prevention. J Clin Exp Hepatol 2024; 14: 101432. 38975605 10.1016/j.jceh.2024.101432 PMC11222954

[19] Stauffer JA, Steers JL, Bonatti H, et al. Liver transplantation and pancreatic resection: a single-center experience and a review of the literature. Liver Transpl 2009; 15: 1728–37. 19938125 10.1002/lt.21932

[20] van Vilsteren FG, Baskin-Bey ES, Nagorney DM, et al. Liver transplantation for gastroenteropancreatic neuroendocrine cancers: defining selection criteria to improve survival. Liver Transpl 2006; 12: 448–56. 16498656 10.1002/lt.20702

